# Edge-Oriented Compressed Video Super-Resolution

**DOI:** 10.3390/s24010170

**Published:** 2023-12-28

**Authors:** Zheng Wang, Guancheng Quan, Gang He

**Affiliations:** School of Telecommunications Engineering, Xidian University, Xi’an 710071, China; jackwu0630@gmail.com (Z.W.); gcquan@stu.xidian.edu.cn (G.Q.)

**Keywords:** compressed video super-resolution, edge-oriented, recurrent structure

## Abstract

Due to the proliferation of video data in Internet of Things (IoT) systems, in order to reduce the data burden, most social media platforms typically employ downsampling to reduce the resolution of high-resolution (HR) videos before video coding. Consequently, the loss of detail and the introduction of additional artifacts seriously compromise the quality of experience (QoE). Recently, the task of compressive video super-resolution (CVSR) has garnered significant attention, aiming to simultaneously eliminate compression artifacts and enhance the resolution of compressed videos. In this paper, we propose an edge-oriented compressed video super-resolution network (EOCVSR), which focuses on reconstructing higher-quality details, to effectively address the CVSR task. Firstly, we devised a motion-guided alignment module (MGAM) to achieve precise bi-direction motion compensation in a multi-scale manner. Secondly, we introduced an edge-oriented recurrent block (EORB) to reconstruct edge information by combining the merits of explicit and implicit edge extraction. In addition, benefiting from the recurrent structure, the receptive field of EOCVSR can be enhanced and the features can be effectively refined without introducing additional parameters. Extensive experiments conducted on benchmark datasets demonstrate that our method surpasses the performance of state-of-the-art (SOTA) approaches in both quantitative and qualitative evaluations. Our approach can provide users with high-quality and cost-effective HR videos by integrating with sensors and codecs.

## 1. Introduction

Nowadays, the explosion of high-resolution (HR) videos has surged with the rapid advancement of portable device sensors and 5G communication technology. Some popular social media platforms, such as TikTok and WeChat, often downsample HR videos before compression to minimize the expenses associated with data storage and transmission, especially when bandwidth and storage space are severely constrained. The decoded low-resolution videos need to be upsampled back to their original resolution to meet user requirements. However, ensuring a satisfactory quality of experience (QoE) is extremely challenging due to the information loss caused by downsampling and the introduction of compression artifacts.

Despite the significant achievements of deep learning techniques in video quality enhancement (VQE) [1,2,3,4,5,6] and video super-resolution (VSR) tasks [7,8,9,10,11], simply cascading two networks to upsample the LR compressed videos in two stages often fail to yield satisfactory results. The main reason is that the information of the two independent processes cannot be effectively collaborated. Recently, there has been a growing interest in the task of compressed video super-resolution (CVSR) [12,13,14], which sought to address both compression artifacts removal and resolution enhancement in compressed videos simultaneously. An end-to-end restoration-reconstruction deep neural network (RR-DnCNN) [12] was first proposed to entirely solve degradation from compression and downsampling. The authors utilized upsampling skip connections to pass the useful features extracted by restoration to reconstruction. He et al. introduced a novel model, named Feature Multiplexing Video Super-Resolution for Compressed Video (FM-VSR) [13], to recover high-quality, high-resolution videos from low-resolution videos compressed with high compression rates. However, these methods lack targeted processing for high-frequency components, particularly edge components. Furthermore, they solely rely on the mean squared error (MSE) loss function during training, resulting in the inadequate reconstruction of details and overly smooth and blurry outputs. There is a strong demand for robust CVSR algorithms that collaborate with sensors and video codecs to produce exceptional HR videos.

To this end, we propose an edge-oriented compressed video super-resolution network (EOCVSR), which pays more attention to reconstructing high-quality details to effectively address the CVSR task. EOCVSR takes the target frame and its two temporally adjacent frames as inputs to fully leverage the information from multiple frames. Firstly, we devised a motion-guided alignment module (MGAM) to achieve precise bi-direction motion compensation. We analyzed the explicit optical flow between two frames in a multi-scale manner to generate an attention map. The offset of the deformable convolution [15] generated under the guidance of the attention map enables accurate implicit temporal alignment. Secondly, we proposed an edge-oriented recurrent block (EORB) to reconstruct edge information. We adopted several conventional filters and some learnable convolution kernels with specific shapes to derive edge information. By combining the merits of explicit and implicit edge extraction, we can perform a more targeted and high-quality reconstruction of high-frequency components. Furthermore, we designed a recurrent structure to enhance the receptive field and the performance of EOCVSR without introducing additional parameters. The edge-oriented loss function is also employed during training to boost performance. Extensive experiments conducted on benchmark datasets demonstrate that our method surpasses the performance of SOTA approaches in both quantitative and qualitative evaluations. The main contributions can be summarized as follows:

- We propose an edge-oriented compressed video super-resolution network (EOCVSR) to address the CVSR problem. By incorporating a structure that specifically processes edge information and introduces edge-related loss functions, EOCVSR is able to reconstruct richer details and output higher-quality frames.
- We propose a motion-guided alignment module (MGAM) to achieve precise bi-direction motion compensation. The utilization efficiency of temporal information is enhanced by employing explicit motion information to guide the generation of offsets for implicit temporal alignment.
- We propose an edge-oriented recurrent block (EORB) to reconstruct edge information. Combining the merits of explicit and implicit edge extraction enables the high-quality reconstruction of high-frequency components. In addition, a recurrent structure is also adopted to realize effective feature refinement.

## 2. Related Works

### 2.1. Video Quality Enhancement (VQE)

With the widespread dissemination of video content and the increasing demand for storage, improving the quality of compressed videos has become a crucial task. Traditional video coding techniques introduce various distortions and compression artifacts during video compression, leading to a degradation in video quality. Consequently, researchers have begun exploring the use of deep learning methods to enhance the visual quality of compressed videos. An early work in the field of deep learning-based elimination of coding artifacts is the Variable-filter-size Residue-learning CNN (VRCNN) [1]. VRCNN integrates convolutional neural networks (CNNs) into the in-loop filter of High-Efficiency Video Coding (HEVC) [16] intra-coding, leading to improved coding performance. Wang et al. introduced a Deep CNN-based Auto-Decoder (DCAD) approach [2], which directly enhances the decoded frames without modifying the specific components of the decoder. Recognizing the distinction between intra-coding and inter-coding in HEVC, Yang et al. proposed the Quality-Enhancement Convolutional Neural Network (QECNN) [3]. QECNN comprises two subnetworks, namely QECNN-I and QECNN-B, which are designed to enhance the compressed I frames and P/B frames, respectively. Due to the lack of utilization of adjacent frames, the enhancement performance of these single-frame methods is limited. Therefore, some multi-frame methods [4,5,6] have been developed to leverage the temporal relationships between frames. Yang et al. observed that frames captured within a short period exhibit high similarity, leading them to propose a multi-frame quality enhancement (MFQE) approach [4]. This approach comprises a peak quality frames (PQFs) detector and a multi-frame convolutional neural network (MF-CNN). Building upon this, MFQE 2.0 [5] introduces an improved PQF-detector based on LSTM and a lightweight architecture for the MF-CNN, aiming to achieve enhanced performance more efficiently. Additionally, a spatio-temporal deformable fusion scheme [6] is employed, utilizing deformable convolutions to leverage temporal information. However, these existing methods need to be followed by an upsampling process to be applied to the CVSR task. The independence of the two processes from each other leads to an incoherent enhancement of the compressed video.

### 2.2. Video Super-Resolution (VSR)

Video super-resolution aims to improve video quality and detail reconstruction by learning the spatial and temporal characteristics of the video and upgrading the low-resolution video to high resolution. VSRnet [7] is an extension of the image super-resolution algorithm SRCNN [17] to the video domain. The major improvement lies in the inclusion of motion estimation and motion compensation modules, where the input is transformed from a single frame to multiple frames. Jo et al. introduced a DUF network [11] that generates dynamic upsampling filters and a residual image, which are computed depending on the local spatio-temporal neighborhood of each pixel to avoid explicit motion compensation. Tian et al. proposed a temporally deformable alignment network (TDAN) [10] to adaptively align the reference frame and each supporting frame with a feature level without computing optical flow. Concurrently, EDVR [8] devised an innovative spatio-temporal attention fusion module, along with enhanced deformable convolutions, to effectively handle motion compensation. However, when applying these methods to the CVSR task, compression artifacts may become more pronounced, significantly impacting video quality.

### 2.3. Compressed Video Super-Resolution (CVSR)

Recently, there has been a growing interest in the task of compressed video super-resolution (CVSR), which seeks to address both compression artifact removal and resolution enhancement in compressed videos simultaneously. An end-to-end restoration-reconstruction deep neural network (RR-DnCNN) [12] was proposed, which uses the degradation-aware technique to effectively deal with video compression distortion and upsampling degradation. In its extended version, RR-DnCNN v2 [14], the authors redesigned the network architecture by adopting a U-shaped form and incorporating upsampling skip connections. This architectural modification enables the propagation of valuable features captured during the restoration process to the subsequent reconstruction stage. He et al. proposed a one-stage video super-resolution network (FM-VSR) [13] to recover high-quality, high-resolution videos from low-resolution videos. They make full use of the internal correlation between the VQE and VSR tasks by adopting a feature multiplexing mechanism. However, these methods exhibit limitations in their treatment of high-frequency components, specifically edge components, as they do not incorporate targeted processing techniques. Additionally, their reliance solely on the mean squared error (MSE) loss function during the training phase leads to suboptimal reconstruction of fine details and outputs that are excessively smooth and blurry.

## 3. The Proposed EOCVSR Approach

### 3.1. Overall Framework

**Figure 1:**
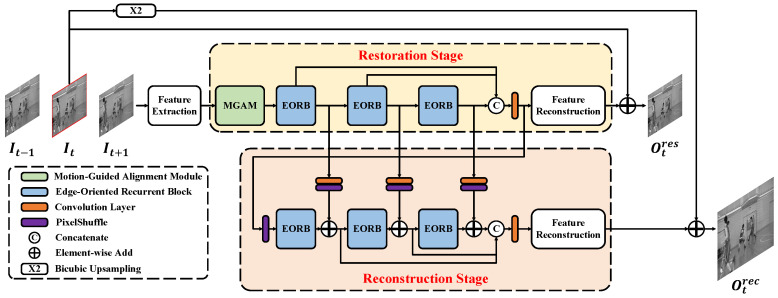
The overall framework of our proposed EOCVSR.

The overall framework of our proposed EOCVSR approach is depicted in Figure 1. EOCVSR takes the target frame $I_{t}$ and its two temporally adjacent frames $I_{t-1}$ and $I_{t+1}$ as the input. First of all, a parameter-shared feature extraction module transforms three input frames from pixel space to feature space. As shown in Figure 1, the following procedure can be divided into two stages: restoration and reconstruction. The restoration stage aims to eliminate the compression artifacts. To make full use of temporal multi-frame information, a motion-guided alignment module is employed to achieve precise bi-direction motion compensation. Then, we stack three edge-oriented recurrent blocks (EORBs) to refine the aligned feature. The intermediate outputs of EORBs in the restoration stage are fused by a convolution layer. The fused feature is fed into a feature reconstruction module to obtain a residual image, which will be added to $I_{t}$ to generate the result of the restoration stage $O_{t}^{res}$.

The reconstruction stage, which takes the fused feature output by the restoration stage as the input, aims to enhance the resolution and reconstruct high-quality details. The pixel-shuffle operation is first adopted to upsample the input feature. Another three cascading EORBs extract edge information at a larger scale. In the reconstruction stage, the output of each EORB is integrated with the output of the EORB, which is in the same order in the restoration stage. Such internal correlation between the two stages improves the learning ability of EOCVSR. Same as in the restoration stage, we use a convolution layer to fuse the outputs of EORBs from the reconstruction stage. Finally, the result of the reconstruction stage $O_{t}^{rec}$ can be obtained by adding the residual image reconstructed from the high-resolution fused feature to the result of upsampling $I_{t}$ using the Bi-cubic operation. The function of our proposed EOCVSR can be formulated as

(1) $$O_{t}^{res},O_{t}^{rec}=\Phi \left(I_{t-1},I_{t},I_{t+1}|\theta \right),$$

where Φ is the proposed approach and θ is the parameters of the network. The symbols that will appear with the corresponding explanations are shown in Table 1.

### 3.2. Feature Extraction Module

The feature extraction module is defined as

(2) $$F_{n}=f_{fe}\left(I_{n}\right),n\in \left\{t-1,t,t+1\right\},$$

where $f_{fe}\left(\cdot \right)$ denotes the feature extraction function, and *F* represents the extracted feature of the input frames *I*. First, a convolution layer (Conv) is used to transform the input frames from a pixel space to a higher dimensional feature space. Then, we adopt a typical residual block (RB) [18] to roughly process the features. The parameters used to process the inputted three frames are shared.

### 3.3. Motion-Guided Alignment Module

**Figure 2:**
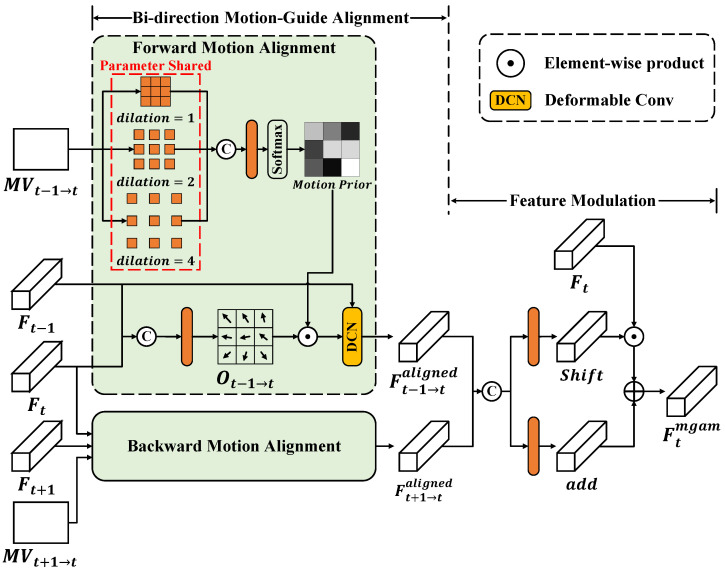
The structure of motion-guided alignment module (MGAM).

The motion-guided alignment module (MGAM) aims to achieve precise bi-direction motion compensation and derive useful temporal multi-frame information for restoration. As shown in Figure 2, the structure of MGAM is divided into two parts, bi-direction motion-guided alignment and feature modulation. In the first part, we utilize explicit motion prior, i.e., optical flow, to guide feature-level temporal alignment. Take the forward alignment as an example, we first obtain the motion vector $MV_{t-1\to t}$ using a pre-trained SpyNet [19], which is a widely used optical flow prediction network.

(3) $$MV_{t-1\to t}=SpyNet\left(I_{t},I_{t-1}\right)$$

Then, we employ a parameter-shared Conv with a different dilation d∈{1,2,4} to implement multi-scale analysis on $MV_{t-1\to t}$. The analyzed results are concatenated, fused, and outputted by a softmax layer, resulting in the generation of a motion prior in the form of an attention map.

(4) $$\left\{\begin{array}{c} h1=Conv{\left(MV_{t-1\to t}\right)}_{d=1},\\ h2=Conv{\left(MV_{t-1\to t}\right)}_{d=2},\\ h3=Conv{\left(MV_{t-1\to t}\right)}_{d=3},\\ MotionPrior=Softmax(Conv([h1,h2,h3])). \end{array},\right.$$

where [·,·] and Softmax are concatenation and the softmax layer. Meanwhile, an offset of the deformable convolution (DCN) kernel $O_{t-1\to t}$ is dynamically predicted from $F_{t}$ and $F_{t-1}$. Before directly applying $O_{t-1\to t}$ to DCN, we fine-tune $O_{t-1\to t}$ by multiplying the motion prior with it, so that it can better capture the motion correlation. By feeding $F_{t-1}$ and the fine-tuned $O_{t-1\to t}$ into a DCN, a forward-aligned feature $F_{t-1\to t}^{aligned}$ can be obtained. The detailed process is given below:

(5) $$\left\{\begin{array}{c} O_{t\to t-1}=Conv\left(\left[F_{t},F_{t-1}\right]\right),\\ F_{t-1\to t}^{aligned}=DCN\left(F_{t-1},O_{t\to t-1}⊙MotionPrior\right). \end{array},\right.$$

where DCN and ⊙ denote the deformable convolution layer and element-wise product. The backward alignment takes $F_{t}$, $F_{t+1}$, and the backward optical flow takes $MV_{t+1\to t}$ as the input and outputs a backward-aligned feature $F_{t+1\to t}^{aligned}$. The processing is mirrored in the forward alignment.

In the second part, we adopt the method of feature modulation to realize the fusion of multi-frame information. $F_{t-1\to t}^{aligned}$ and $F_{t+1\to t}^{aligned}$ are first concatenated together, and then the concatenation result is fed into two Convs to generate the shift and add weights for modulation, respectively. This operation can be described as

(6) $$\left\{\begin{array}{c} shift=Conv(\left[F_{t-1\to t}^{aligned},F_{t+1\to t}^{aligned}\right]),\\ add=Conv(\left[F_{t-1\to t}^{aligned},F_{t+1\to t}^{aligned}\right]),\\ F_{t}^{mgam}=F_{t}⊙shift+add. \end{array}.\right.$$

Thanks to precise motion alignment in the MGAM, the aligned features provide significant improvement to the overall performance.

### 3.4. Edge-Oriented Recurrent Block

**Figure 3:**
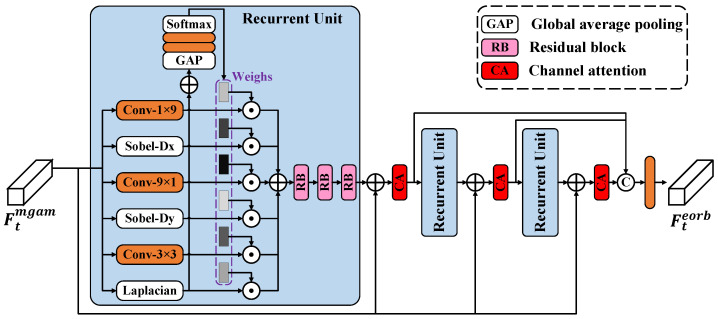
The structure of edge-oriented recurrent block (EORB).

Most existing CVSR approaches lack targeted processing for high-frequency components, particularly edge components. Furthermore, they solely rely on mean squared error (MSE) loss function during training, resulting in inadequate reconstruction of details and overly smooth and blurry outputs. To this end, we devise an edge-orient recurrent block (EORB) to augment the network’s capacity for perceiving and reconstructing details. As shown in Figure 3, the recurrent unit is the key component of the EORB. The input of the recurrent unit is first processed by six different filters for edge-aware. To perceive horizontal edges, we adopt a horizontal Sobel filter and a learnable Conv with the size of 1×9. To perceive vertical edges, we adopt a vertical Sobel filter and another learnable 9×1 Conv. We also extract the second-order spatial derivative using a Laplacian filter, collaborating with a learnable 3×3 Conv. Combining the merits of pre-defined and learnable edge filters, the edge information can be efficiently derived. It is worth noting that the shapes of the six detectors will not change, and the parameters of the three learnable edge detectors can be updated through the back-propagation operation. Then, the summation of all the outputs, followed by the application of a global average pooling (GAP) layer, two Convs, and a softmax layer, generates the weights corresponding to each output. By multiplying the outputs with their corresponding weights and subsequently accumulating them, valuable edge information is filtered and preserved. The process within the recurrent unit is described as

(7) $$\left\{\begin{array}{c} O_{1}=Conv_{9\times 1}\left(F_{t}^{RU-k}\right),O_{2}=Sobel_{Dx}\left(F_{t}^{RU-k}\right)\\ O_{3}=Conv_{1\times 9}\left(F_{t}^{RU-k}\right),O_{4}=Sobel_{Dy}\left(F_{t}^{RU-k}\right)\\ O_{5}=Conv_{3\times 3}\left(F_{t}^{RU-k}\right),O_{6}=Laplacian\left(F_{t}^{RU-k}\right)\\ W_{1}∼W_{6}=Softmax(Conv\left(Conv\left(GAP\left(\sum_{i=1}^{6}O_{i}\right)\right)\right)),\\ RU\left(\cdot \right)=Conv\left(\sum_{i=1}^{6}O_{i}⊙W_{i}\right) \end{array},\right.$$

where RU(·) represents the process of the recurrent unit. k∈[1,K−1] stands for the current iteration of recursion and *K* is the maximum number of recursions. At last, we stack three RBs to further process the feature. The output of the previous RB is utilized as the input of the next RB. Only the output of the last RB is added to the input feature of the EORB, which is $F_{t}^{mgam}$.

To enhance the receptive field and performance without introducing additional parameters, we adopt a recurrent strategy. For the current recursion, the output of the previous recursion is added to the input of the EORB $F_{t}^{input}$ and is subsequently passed through a channel attention layer. No matter how many times the recursion is performed during the training, it still belongs to the forward inference phase and does not involve updating the parameters. Therefore, in each recursion, the parameters of the recurrent unit are shared. The output of the EORB $F_{t}^{eorb}$ is obtained by using a Conv to fuse the concatenation result of the outputs of all recursions. The detailed process is given below:

(8) $$\left\{\begin{array}{c} F_{t}^{RU-k}=CA\left(RU\left(F_{t}^{input}\right)+F_{t}^{input}\right),\\ F_{t}^{RU-(k+1)}=CA\left(RU\left(F_{t}^{RU-k}\right)+F_{t}^{input}\right)\\ F_{t}^{eorb}=Conv\left(\left[F_{t}^{RU-1},F_{t}^{RU-(k+1)}\right]\right). \end{array}.\right.$$

### 3.5. Feature Reconstruction Module

The feature reconstruction module takes the fused result of the outputs of all previous EORBs as the input. The feature reconstruction module consists of an RB and a Conv, which outputs a residual image $R_{t}$. The process is defined as

(9) $$R_{t}=f_{fr}\left(Conv\left(\left[F_{t}^{eorb-M}\right]\right)\right),$$

where $f_{fr}\left(\cdot \right)$ is the feature reconstruction function. *M* is the number of EORBs of each stage. In the restoration stage, the residual image will be added to $I_{t}$ to obtain $O_{t}^{res}$. As for the reconstruction stage, the residual image will be added to the upsampling result of $I_{t}$ to obtain $O_{t}^{rec}$. It is worth mentioning that the feature reconstruction modules in the restoration and reconstruction stages use different parameters.

### 3.6. Loss Function

Firstly, in addition to calculating the reconstruction error between the super-resolution result $O_{t}^{rec}$ and the uncompressed HR frame $G_{t}^{HR}$, we also use the uncompressed LR frame $G_{t}^{LR}$ as an auxiliary supervised label to assist the network training. Thus, the total loss function is defined as

(10) $$L_{total}=\alpha *L_{res}+L_{rec},$$

where α is the weighted factor for the restoration loss.

However, only relying on the mean squared error (MSE) loss function during training may cause inadequate reconstruction of details and overly smooth and blurry outputs. To this end, we introduce an edge-orient loss function to direct constraints on the learning of edge reconstruction. Specifically, we explicitly extract high-frequency components using a Gaussian kernel blur g(·,·) with the width of σ. The details of the loss function are shown below:

(11) $$\begin{array}{cc} L_{res} & =L_{mse}\left(O_{t}^{res},G_{t}^{LR}\right)+\beta *L_{mse}\left(OE_{t}^{res},GE_{t}^{LR}\right)\\ L_{rec} & =L_{mse}\left(O_{t}^{rec},G_{t}^{HR}\right)+\beta *L_{mse}\left(OE_{t}^{rec},GE_{t}^{HR}\right)\\ OE_{t} & =O_{t}-g\left(O_{t},\sigma =3\right)\\ GE_{t} & =G_{t}-g\left(G_{t},\sigma =3\right) \end{array}.$$

## 4. Results

### 4.1. Experimental Setup

To train our proposed EOCVSR, we use the Vimeo dataset [19], which contains about 65,000 video sequences. Each sequence has seven frames with 448×256 resolution. We first use Bi-cubic interpolation to obtain uncompressed LR videos by a downsampling factor of two. Then, we compress these LR videos using FFmpeg [20] with the default mode and CRFs = 32, 37, 42, and 47. Finally, the bitstream is decoded to generate the compressed LR videos. To evaluate the trained EOCVSR, we adopt the test sequences from the standard HEVC common test condition (CTC) [21], the UVG dataset [22], and the MCL-JCV dataset [23]. The downsampling and compression settings are consistent with those in the training.

During the training, we randomly crop 120×120 patches from a mini-batch as the input. The batch size is set as eight. The hyperparameters regarding the network structure *K* and *M* are both set as three. The model trained with the loss function described in Section 3.5, and the weights α and β, are set as 0.2 and 0.1, respectively. The learning rate is initialized as $1\times 10^{-4}$ and then divided by a factor of 10 every 30 epochs. The training stops after 100 epochs. The Adam optimizer [23] is used by setting $\beta_{1}=0.9$ and $\beta_{2}=0.999$. During the evaluation, we use BD-BR [24], which presents the quality improvement (dB) at the same bitrate, and PSNR for quantitative analysis of the compressed video super-resolution results. All the models are implemented with PyTorch 1.4.0 [25] on Nvidia Geforce 2080Ti GPUs. All calculations are on the luminance channel (Y channel).

### 4.2. Performance of Proposed EOCVSR

The performance of our proposed EOCVSR is compared with the latest CVSR approaches, including RR-DnCNN v2 [14] and FM-VSR [13]. As shown in Table 2, we compare the characteristics between EOCVSR and other CVSR approaches. We also retrain some advanced video super-resolution approaches, including [8] and BasicVSR++ [9], over our training dataset for comparison. The comparison of the size of each model and computation cost is displayed in Table 3. We evaluate the quality enhancement, the rate–distortion performance, and subjective performance. The details are described as follows.

#### 4.2.1. Quality Enhancement

Table 4 presents the PSNR results over HEVC standard test sequences. First, the results illustrate that the proposed EOCVSR outperforms all the prior approaches over four compression ratios. Specifically, the PSNR of EOCVSR is 29.037 dB, which is 0.038 dB higher than the state-of-the-art approach, i.e., BasicVSR++ (28.999 dB), and 0.107–0.325 dB higher than others [8,13,14]. Table 5 shows the PSNR results over the UVG and MCL-JCV datasets, and the results demonstrate that EOCVSR also achieves the most significant boost. In terms of the UVG dataset, the PSNR improvement of EOCVSR ranges from 0.022 to 0.140. In terms of the MCL-JCV dataset, the PSNR improvement of EOCVSR ranges from 0.031 to 0.200. Thanks to MGAM’s precise motion alignment and EORM’s powerful edge information extraction and reconstruction capabilities, our proposed EOCVSR achieves the highest quality enhancement over all evaluation datasets.

#### 4.2.2. Rate–Distortion Performance

**Figure 4:**
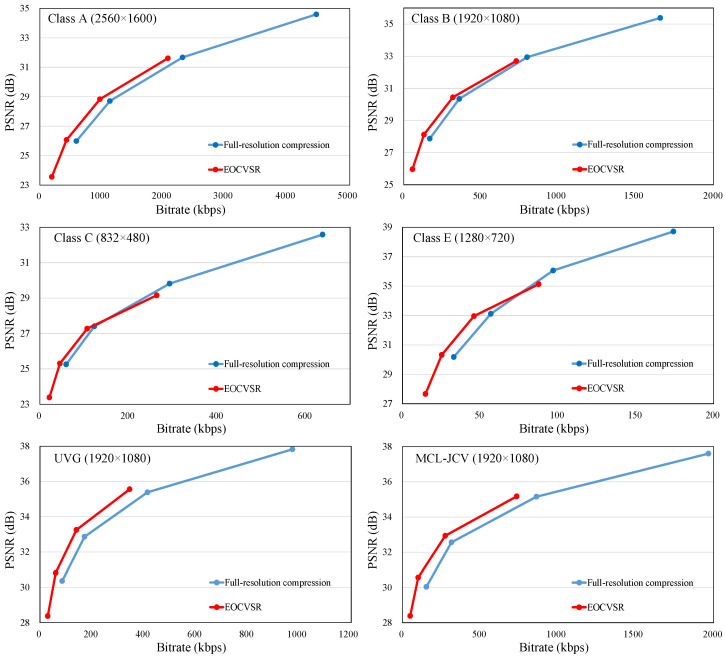
The RD curve over all test datasets of various resolutions under different compression distortion conditions.

Here, we evaluate the rate–distortion of EOCVSR over three datasets, and the results are shown in Table 6. Considering full-resolution compression as an anchor, the performance has been improved in terms of 14.364% BD-BR reduction on average over HEVC standard test sequences, which is 0.843% more than BasicVSR++ (13.521%), and 2.737–8.277% more than other approaches [8,13,14]. As for the UVG and the MCL-JCV datasets, the bitrate saving achieves 29.849% and 28.217%. To present more intuitively, rate–distortion curves of our and other approaches over all test sequences are shown in Figure 4. In this figure, we can observe that the curve of EOCVSR is above that of full-resolution compression. In brief, the quantitative results demonstrate that our proposed EOCVSR realizes the best compression performance.

#### 4.2.3. Subjective Performance

**Figure 5:**
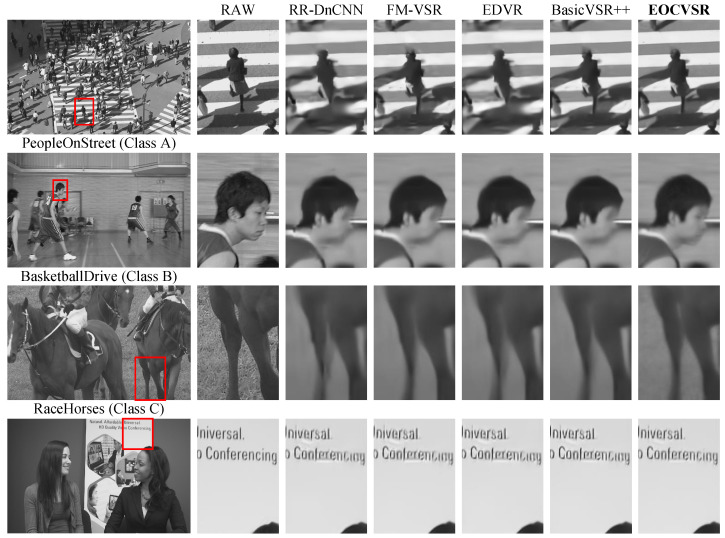
Visual comparisons of different methods on video frames from HEVC standard test sequences (QP = 42). The zoom-in of red box area is shown.

Figure 5 shows the visual results of the different methods. Observing the results, it is evident that our proposed EOCVSR has demonstrated substantial advancements in visual perception when compared to other approaches. The compression artifacts are effectively eliminated. Thanks to the excellent edge-awareness of EOCVSR, the edges and textures are reconstructed with high quality. For example, the face in the sequence BasketballDrive and the letters in the sequence KristenAndSara. Therefore, our EOCVSR approach achieves promising performance in subjective quality.

### 4.3. Ablation Study

In this section, we conduct several ablation experiments to analyze the impact of different structures of the proposed EOCVSR on performance. All the experiments are performed over the MCL-JCV dataset and the compression ratio CRF is set as 47. In general, the results demonstrate that EOCVSR is delicately designed to achieve optimal performance. The details are described as follows.

#### 4.3.1. Analysis of the EORB

**Figure 6:**
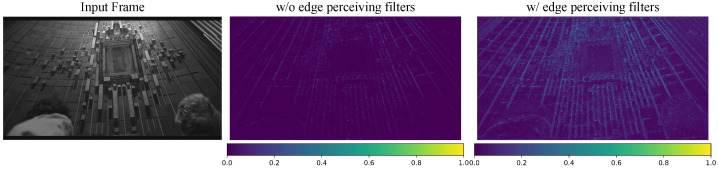
The feature map output by EORB with edge-perceiving filters vs. without edge-perceiving filters.

An ablation study has been conducted to verify the effectiveness of our proposed EORB. We modify the EORB by removing the six filters used to perceive the edges and leaving only the RBs. As shown in Table 7, the original EORB achieves a 28.386 dB improvement in PSNR, while the performance of the modified EORB degrades to 28.317 dB. The results prove that combining the merits of pre-defined and learnable edge filters enables EOCVSR to efficiently extract the edge information for higher performance. Furthermore, we provide a feature map visualization example. As shown in Figure 6, the original EORB is more sensitive to edges and textures.

#### 4.3.2. Analysis of the Number of Recursions *K*

**Figure 7:**
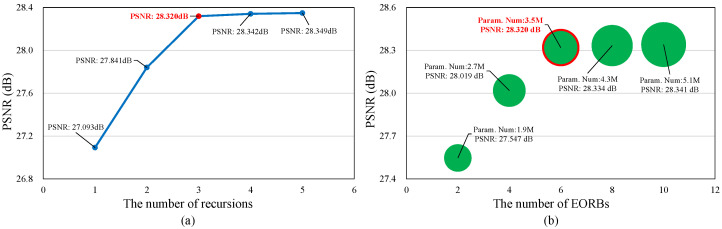
Ablation study on (**a**) the number of recursions within an EORB and (**b**) the number of EORBs.

We design a recurrent structure to enhance the receptive field and the performance of EOCVSR without introducing additional parameters, and the number of recursions within an EORB has an impact on the performance of EOCVSR. As shown in Figure 7a, the performance increases quickly when *K* is from one to three, while the performance increases slowly when *K* is larger than three. Considering more recursions may extend the processing time, *K* is set as three in this work.

#### 4.3.3. Analysis of the Number of EORBs *M*

We evaluate EOCVSR with different numbers of EORBs. Integrating more EORBs can enhance the representation capabilities of the network but introduce more parameters. Figure 7b shows the performance of quality enhancement versus the number of EORBs *M*. The performance gain increases slowly when *M* is from 6 to 10. Thus, *M* is set as six (three in the restoration stage and another three in the reconstruction stage) in this work.

#### 4.3.4. Model Adaption

We evaluate the adaptability of the proposed EOCVSR to different types of tasks. Theoretically, the proposed design can be applied to many other video tasks, such as denoising tasks, enhancement tasks, deblurring tasks, and so on. We add Gaussian noise to the image frames to train and test the ability of EOCVSR to perform super-resolution denoising at noise levels of σ = 15, 25, and 50. The parameter settings and network training are the same as for the original task. We compared the performance of EOCVSR with MIRNet [26]. As shown in Table 8, the PSNR of EOCVSR is improved by 0.45–1.4 dB. The results show that our method outperforms the learning-based super-resolution denoising method, illustrating that our model possesses the ability to adapt to different types of tasks.

## 5. Conclusions

In this paper, we proposed an edge-oriented compressed video super-resolution network (EOCVSR), which pays more attention to reconstructing high-quality details to effectively address the CVSR task. We devised a motion-guided alignment module (MGAM), which uses the explicit optical flow to direct the generation of offsets for deformable convolutions leading to precise bi-direction motion compensation. In addition, we proposed an edge-oriented recurrent block (EORB) to reconstruct edge information. Several pre-defined filters and some learnable convolution kernels with specific shapes were exploited to derive edge information. By combining the merits of explicit and implicit edge extraction, we can perform a more targeted and high-quality reconstruction of high-frequency components. We also designed a recurrent structure to enhance the receptive field and the performance of EOCVSR without introducing additional parameters. Our approach can provide users with high-quality and cost-effective HR videos by integrating with sensors and codecs.

## Figures and Tables

**Table 1:** List of symbols.

Symbols	Explanation
$I_{t}$	Input video frame *t*
$O_{t}^{res}$	Video restoration output
$O_{t}^{rec}$	Video reconstruction output
$F_{t}$	The feature of frame *t*
MV	Motion vector
*O*	The offset of the deformable convolution kernel
$F^{aligned}$	The aligned feature
$F_{t}^{mgam}$	The output feature of MGAM for frame *t*
$Conv_{m\times n}$	Convolutional layer with kernel of m×n
Softmax	Softmax normalized activation function
$Sobel_{Dx}$	Vertical edge detection operator of Sobel
$Sobel_{Dy}$	Horizontal edge detection operator of Sobel
Laplacian	Laplacian edge detection operator
⨀	Element-wise product
⨁	Element-wise add
$G_{t}^{LR}$	Low-resolution Ground Truth at frame *t*
$G_{t}^{HR}$	High-resolution Ground Truth at frame *t*
$L_{mse}$	Mean squared error loss function

**Table 2:** Comparison with previous CVSR works.

Approach	Multi-Frame Utilization	Edge Preservation
RR-DnCNN v2	×	×
FM-VSR	✓	×
EOCVSR (proposed)	✓	✓

**Table 3:** The comparison of the number of model parameters and GFLOPs. GFLOPs are calculated on an image with an input size of 64 × 64.

	RR-DnCNN v2	FM-VSR	EDVR	BasicVSR++	EOCVSR
Parameter Number	1.8M	7.1M	2.7M	7.1M	3.5M
GFLOPs	20.9	96.1	66.6	104.3	88.4

**Table 4:** The comparison of PSNR gain over HEVC standard test sequences. Red indicates the best performance, and blue indicates the second-best.

QP	Class	Sequences	RR-DnCNN v2	FM-VSR	EDVR	BasicVSR++	EOCVSR
32	A	PeopleOnStreet	29.608	29.813	28.859	30.029	**30.129**
		Traffic	32.817	32.866	32.976	33.069	**33.102**
	B	BasketballDrive	31.726	31.902	32.422	32.471	**32.531**
		Cactus	31.012	31.297	31.566	31.712	**31.735**
		Kimono	34.456	34.735	34.799	34.844	**34.876**
		ParkScene	31.229	31.384	31.608	31.655	**31.671**
	C	BasketballDrill	30.177	30.379	30.460	30.636	**30.706**
		RaceHorses	24.544	26.111	27.524	27.599	**27.609**
	E	FourPeople	33.757	33.865	33.929	34.103	**34.260**
		Johnny	35.705	35.926	36.101	36.137	**36.162**
		KristenAndSara	34.282	34.510	34.660	34.919	**34.973**
	**Average**		32.017	32.072	32.348	32.470	**32.523**
37	**Average**		29.962	30.093	30.137	30.210	**30.262**
42	**Average**		27.559	27.610	27.775	27.851	**27.838**
47	**Average**		25.308	25.366	25.459	25.464	**25.524**
**Overall**			28.712	28.785	28.930	28.999	**29.037**

**Table 5:** The comparison of average PSNR gain over the UVG and MCL-JCV datasets. Red indicates the best performance, and blue indicates the second-best.

Dataset	QP	RR-DnCNN v2	FM-VSR	EDVR	BasicVSR++	EOCVSR
UVG	32	35.352	35.358	35.450	35.526	**35.558**
	37	33.142	33.167	33.151	33.237	**33.250**
	42	30.683	30.701	30.755	30.822	**30.822**
	47	28.267	28.243	28.326	28.331	**28.375**
	**Overall**	31.861	31.867	31.920	31.979	**32.001**
MCL-JCV	32	34.860	34.935	35.053	35.140	**35.172**
	37	32.748	32.749	32.774	32.890	**32.930**
	42	30.426	30.480	30.500	30.575	**30.568**
	47	28.222	28.304	28.313	28.328	**28.386**
	**Overall**	31.564	31.617	31.660	31.733	**31.764**

**Table 6:** The comparison of BD-BR over HEVC standard test sequences. Red indicates the best performance, and blue indicates the second-best.

Dataset	Class	Sequences	RR-DnCNN v2	FM-VSR	EDVR	BasicVSR++	EOCVSR
HEVC	A	PeopleOnStreet	−11.621	−13.780	−15.541	−18.113	**−20.621**
		Traffic	−3.916	−5.019	−6.153	−7.833	**−8.584**
	B	BasketballDrive	0.687	−7.133	−11.365	−14.361	**−14.140**
		Cactus	−0.224	−9.965	−12.462	−14.842	**−15.386**
		Kimono	−13.743	−17.421	−20.907	−22.182	**−22.501**
		ParkScene	5.323	−4.408	−7.121	−8.035	**−8.452**
	C	BasketballDrill	0.307	−3.147	−4.883	−6.685	**−8.300**
		RaceHorses	−6.599	−7.366	−8.185	−10.610	**−11.420**
	E	FourPeople	−11.528	−12.154	−12.788	−13.591	**−14.836**
		Johnny	−19.018	−19.580	−20.076	−21.614	**−22.046**
		KristenAndSara	−7.181	−7.877	−8.414	−10.864	**−11.721**
	**Average**		−6.137	−9.804	−11.627	−13.521	**−14.364**
UVG	**Average**		−27.086	−27.313	−27.804	−29.502	**−29.849**
MCL-JCV	**Average**		−24.066	−24.526	−24.913	−27.599	**−28.217**

**Table 7:** The performance of the EORB with edge-perceiving filters vs. without edge-perceiving filters.

	EORB w/o Edge-Perceiving Filters	EORB w/ Edge-Perceiving Filters
PSNR (dB)	28.317	28.386

**Table 8:** The adaption of the proposed design on video super-resolution denoising task.

Noise Level	Scale	MIRNet	EOCVSR
σ=15	×2	34.65	35.10
σ=25	×2	33.86	34.45
σ=50	×2	31.06	32.46

## Data Availability

The data presented in this study are available on request from the corresponding author.

## References

[1] Dai Y., Liu D., Wu F. (2017). A convolutional neural network approach for post-processing in HEVC intra coding. Proceedings of the MultiMedia Modeling: 23rd International Conference, MMM 2017.

[2] Wang T., Chen M., Chao H. A novel deep learning-based method of improving coding efficiency from the decoder-end for HEVC. Proceedings of the 2017 Data Compression Conference (DCC).

[3] Pan Z., Yi X., Zhang Y., Jeon B., Kwong S. (2020). Efficient in-loop filtering based on enhanced deep convolutional neural networks for HEVC. IEEE Trans. Image Process..

[4] Yang R., Xu M., Wang Z., Li T. Multi-frame quality enhancement for compressed video. Proceedings of the IEEE Conference on Computer Vision and Pattern Recognition.

[5] Guan Z., Xing Q., Xu M., Yang R., Liu T., Wang Z. (2019). MFQE 2.0: A new approach for multi-frame quality enhancement on compressed video. IEEE Trans. Pattern Anal. Mach. Intell..

[6] Deng J., Wang L., Pu S., Zhuo C. Spatio-temporal deformable convolution for compressed video quality enhancement. Proceedings of the AAAI Conference on Artificial Intelligence.

[7] Kappeler A., Yoo S., Dai Q., Katsaggelos A.K. (2016). Video super-resolution with convolutional neural networks. IEEE Trans. Comput. Imaging.

[8] Wang X., Chan K.C., Yu K., Dong C., Change Loy C. Edvr: Video restoration with enhanced deformable convolutional networks. Proceedings of the IEEE/CVF Conference on Computer Vision and Pattern Recognition Workshops.

[9] Chan K.C., Zhou S., Xu X., Loy C.C. Basicvsr++: Improving video super-resolution with enhanced propagation and alignment. Proceedings of the IEEE/CVF Conference on Computer Vision and Pattern Recognition.

[10] Tian Y., Zhang Y., Fu Y., Xu C. Tdan: Temporally-deformable alignment network for video super-resolution. Proceedings of the IEEE/CVF Conference on Computer Vision and Pattern Recognition.

[11] Jo Y., Oh S.W., Kang J., Kim S.J. Deep video super-resolution network using dynamic upsampling filters without explicit motion compensation. Proceedings of the IEEE Conference on Computer Vision and Pattern Recognition, Salt Lake City.

[12] Ho M.M., He G., Wang Z., Zhou J. (2020). Down-sampling based video coding with degradation-aware restoration-reconstruction deep neural network. Proceedings of the MultiMedia Modeling: 26th International Conference, MMM 2020.

[13] He G., Wu S., Pei S., Xu L., Wu C., Xu K., Li Y. (2021). FM-VSR: Feature Multiplexing Video Super-Resolution for Compressed Video. IEEE Access.

[14] Ho M.M., Zhou J., He G. (2021). RR-DnCNN v2. 0: Enhanced restoration-reconstruction deep neural network for down-sampling-based video coding. IEEE Trans. Image Process..

[15] Dai J., Qi H., Xiong Y., Li Y., Zhang G., Hu H., Wei Y. Deformable convolutional networks. Proceedings of the IEEE International Vonference on Vomputer Vision.

[16] Sullivan G.J., Ohm J.R., Han W.J., Wiegand T. (2012). Overview of the high efficiency video coding (HEVC) standard. IEEE Trans. Circuits Syst. Video Technol..

[17] Dong C., Loy C.C., Tang X. (2016). Accelerating the super-resolution convolutional neural network. Proceedings of the Computer Vision–ECCV 2016: 14th European Conference.

[18] Lim B., Son S., Kim H., Nah S., Mu Lee K. Enhanced deep residual networks for single image super-resolution. Proceedings of the IEEE Conference on Computer Vision and Pattern Recognition Workshops.

[19] Ranjan A., Black M.J. Optical flow estimation using a spatial pyramid network. Proceedings of the IEEE Conference on Computer Vision and Pattern Recognition.

[20] Newmarch J., Newmarch J. (2017). Ffmpeg/libav. Linux Sound Program..

[21] Bossen F. (2013). Common test conditions and software reference configurations. JCTVC-L1100.

[22] Mercat A., Viitanen M., Vanne J. UVG dataset: 50/120fps 4K sequences for video codec analysis and development. Proceedings of the 11th ACM Multimedia Systems Conference.

[23] Wang H., Gan W., Hu S., Lin J.Y., Jin L., Song L., Wang P., Katsavounidis I., Aaron A., Kuo C.C.J. MCL-JCV: A JND-based H. 264/AVC video quality assessment dataset. Proceedings of the 2016 IEEE International Conference on Image Processing (ICIP).

[24] Grois D., Marpe D., Mulayoff A., Itzhaky B., Hadar O. Performance comparison of h. 265/mpeg-hevc, vp9, and h. 264/mpeg-avc encoders. Proceedings of the 2013 Picture Coding Symposium (PCS).

[25] Imambi S., Prakash K.B., Kanagachidambaresan G. (2021). PyTorch. Programming with TensorFlow: Solution for Edge Computing Applications.

[26] Zamir S.W., Arora A., Khan S., Hayat M., Khan F.S., Yang M.H., Shao L. (2020). Learning Enriched Features for Real Image Restoration and Enhancement. arXiv.

